# Evaluating the Influence of Motor Control on Selective Attention through a Stochastic Model: The Paradigm of Motor Control Dysfunction in Cerebellar Patient

**DOI:** 10.1155/2014/162423

**Published:** 2014-02-09

**Authors:** Giacomo Veneri, Antonio Federico, Alessandra Rufa

**Affiliations:** ^1^Eye Tracking & Visual Application Lab, University of Siena, Viale Bracci 2, 53100 Siena, Italy; ^2^Department of Neurological Sciences and Behavior, University of Siena, Viale Bracci 2, 53100 Siena, Italy

## Abstract

Attention allows us to selectively process the vast amount of information with which we are confronted, prioritizing some aspects of information and ignoring others by focusing on a certain location or aspect of the visual scene. Selective attention is guided by two cognitive mechanisms: saliency of the image (bottom up) and endogenous mechanisms (top down). These two mechanisms interact to direct attention and plan eye movements; then, the movement profile is sent to the motor system, which must constantly update the command needed to produce the desired eye movement. A new approach is described here to study how the eye motor control could influence this selection mechanism in clinical behavior: two groups of patients (SCA2 and late onset cerebellar ataxia LOCA) with well-known problems of motor control were studied; patients performed a cognitively demanding task; the results were compared to a stochastic model based on Monte Carlo simulations and a group of healthy subjects. The analytical procedure evaluated some energy functions for understanding the process. The implemented model suggested that patients performed an optimal visual search, reducing intrinsic noise sources. Our findings theorize a strict correlation between the “optimal motor system” and the “optimal stimulus encoders.”

## 1. Introduction

**Figure 1:**
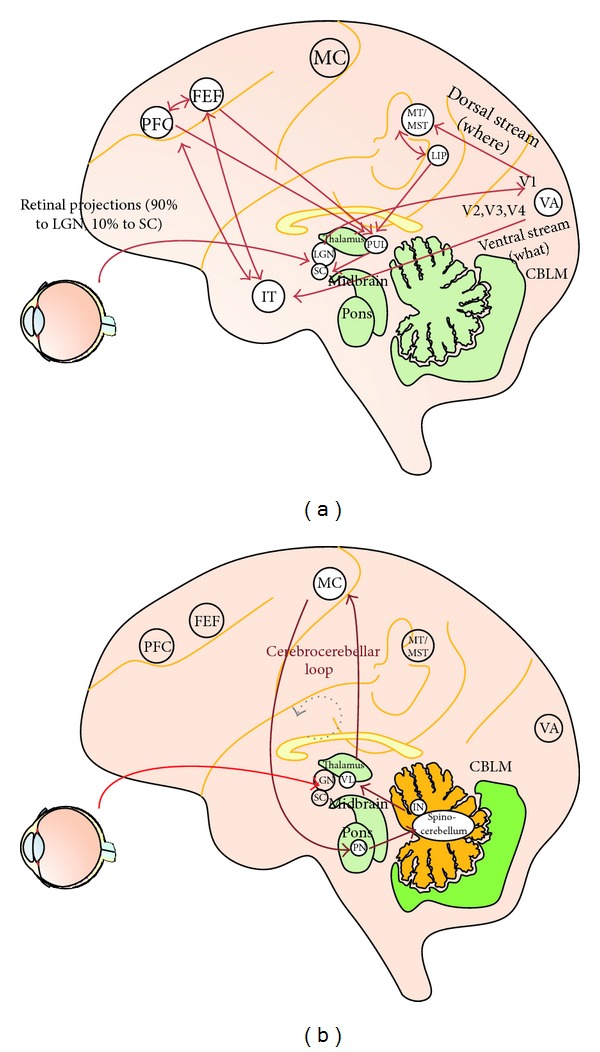
Short reference of vision pathway. (a) Selective attention pathway: the LGN, the striate and extrastriate cortex (V1, V4, inferotemporal cortex, and MT), SC, pulvinar, LIP, FEF, and PFC are known to be involved in attentional processes. (b) Cerebrocerebellar communication loop: the neuronal circuitry of the cerebellum is thought to encode internal models that reproduce the dynamic properties of body parts. These models control the movement allowing the brain to precisely control the movement without the need for sensory feedback. MC: motor cortex. IN: interpositus nucleus. PN: pons. VL: ventrolateral nucleus. SC: superior colliculi. LGN: lateral geniculate nuclei. Visual areas (VA): V1, V2, V3, V4, and MT (middle temporal). PFC: prefrontal cortex. FEF: frontal eye fields. LIP: lateral intraparietal cortex.

The human vision system is a foveocentric structure reflecting the specific anatomical distribution of photoreceptors across the retina, which ensure the best resolution just in a small central region called fovea; outside this region, visual resolution decreases sharply. To overcome this perceptive limit, the human brain has developed fast and accurate eye movements (saccades) for pointing the fovea at interesting objects in space [1]. In other words, each saccade landing point represents the locus in space where the fovea gets the most detailed information; outside this point, elements of a scene may be localized but are less accurately distinguished. Due to this physiological constraint, the amount of information that can be processed at once by visual system is limited; therefore, spatial attention is used to select relevant locations of the visual field for enhanced processing [2] that may occur overtly when associated with an eye movement toward the selected location or covertly without an eye movement. While overt attention is strictly related to saccade motor programming and focuses on the saccade goal, covert attention is more linked to the perceptual characteristics of the peripheral vision and is under the influence of stimulus features (bottom up attraction) and cognitive enhancement (top down conduction). It has been found that visual search of complex scenes is influenced by both top-down factors [3] including previous knowledge, expectations, current cognitive status, and expected goals and bottom-up factors that reflect sensory features of the stimulus such as orientation, luminance, shape, and brightness. In particular bottom up driving of gaze toward the most salient stimuli occurs first; then as visual exploration goes along; there is an increment of cognitive processes influencing visual search in a top down modality. By combining the top down and bottom up information during search, our brain gets a clear view of the conspicuous items (both in terms of cognitive relevance and feature saliency) and their location becoming able to build up an internal, task specific, representation of the scene [4–6]. More specifically the relative conspicuity of each element of the scene is reproduced into a saliency map, which allows to predict where the eyes will be attracted first during the exploration of a scene. Although the saliency map simply refers to the bottom up characteristic of a scene, a more top-down influence of the visual search is represented in the priority map [7]. Covert attention is particularly efficient during top-down visual search where it is used to collect global information of the scene throughout a parallel processing of conspicuous elements and to increase the discrimination abilities of peripheral vision by enhancing spatial resolution at the attended target location [8] and by fading irrelevant locations. From a neuro hysiological point of view, object's features and spatial localizations acquired during visual exploration through the retina [9] are principally sent to visual cortex (V1) via lateral geniculate nucleus (LGN) ([10–12], for a review). However fast spatial information about the visual scene is also sent to the superficial layer of the superior colliculi (SC). This subcortical structure, integrating multisensory inputs with eye and head motor plan, is important for orienting attention, in retinotopic coordinates, towards newly appearing objects in the visual field [10, 13]. More upstreaming, information about object's features and spatial localization is elaborated in the dorsal (occipitotemporal: *where*) and ventral (occipitoparietal: *what*) streams [14–17] in order to construct a priority map [7, 10, 18, 19] (see Figure 1(a)).

Various formal models have been proposed: Feature Integration Theory [20], Guided Search [21], Premotor Theory [22], Theory of Visual Attention [23], and Winner Takes All [24]. Feature Integration Theory proposes a two-stage visual attention process: during first stage humans process several primary visual features; during second stage, the objects are analyzed with details. Winner Takes All and Guided Search are devoted to assign saliency to locations in the visual field. Recently, Reynolds and Heeger [18] proposed a review of a normalized model of attention; the model studied how stimulus features and cognitive attention should work to perform an efficient visual search.

These models describe how humans could select some aspects of the scene, but how the motor control system may optimize visual search by predicting the sensory consequences of an impending saccade? Eye movements are controlled by the cerebrocerebellar communication loop and described by feedforward or inverse control models [25–27] (see Figure 1(b)).

Recently, some authors [28, 29] have applied the theory of optimal control (OCT) in the mechanisms that regulate motor control; humans seem to adapt their behavior to minimize some cost function, such as noise, in performing an action [30]. Najemnik and Geisler argued that humans could choose fixations that maximize information gained about the target's location; this strategy [31] allows humans to select features from the scene optimally such as an “ideal searcher.”

Our work aims to extend the OCT principle to the mechanisms that regulate visual search. We proposed a method to study how the strategies of selective attention may be modulated by the motor control performance. Since the cerebellum is the brain structure where some dynamic aspects of motor control are optimized to reduce errors, we compare our formal model with the ocular motor behavior of well characterized cerebellar patients who have specific motor control failure [32, 33].

**Figure 2:**
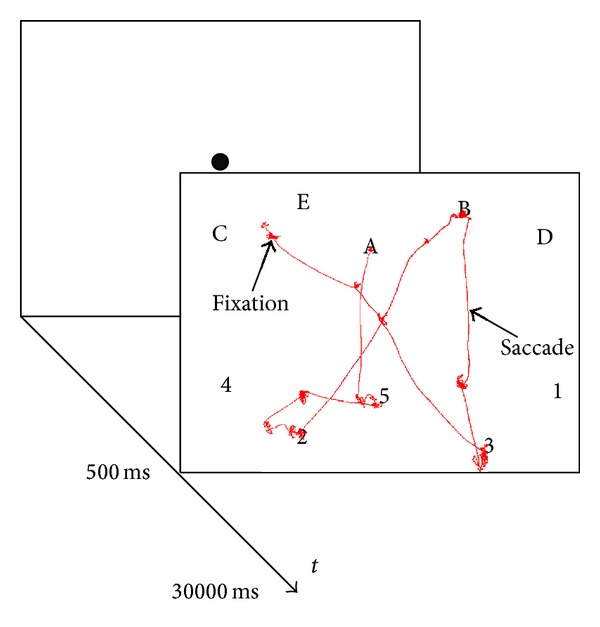
Example of a display shown in the task and a small portion of gaze data (red line). Subjects, after a fixation point of 500 ms, were asked to follow an alphanumeric sequence (1-*A*-2-*B*-3-*C*-4-*D*-5-*E*) with their gaze. The numbers and letters appeared in a pseudorandom distribution (letters on top and numbers on the bottom) on a 1024 × 768 px and 51 × 31 cm black screen for 30000 ms. The red line shows some fixations and saccades made by a healthy subject. Saccades are rapid eye jumps from one region to another region. During fixations (time duration ≈100 ms ⋯ 800 ms) humans acquire information from objects.

Therefore, we developed a mathematical stochastic model based on the Monte Carlo method (MC), able to simulate ongoing visual search in a cognitively demanding task (Figure 2). Some common energy functions were evaluated and we compared model results with a control group of healthy subjects (CTRL) and two groups of patients with degenerative cerebellar ataxia. Indeed, the cerebellum is implicated in maintaining the saccadic subsystem efficient for vision; this ability is often disrupted in degenerative cerebellar diseases, as demonstrated by saccade kinetic abnormalities.

Therefore, two groups of patients affected by spinocerebellar ataxia type 2 (SCA2) and patients with late onset cerebellar ataxia (LOCA) were enrolled in the study. The results of subjects (CTRL, SCA2, and LOCA) were compared with the model's outcome.

## 2. Material and Methods

### 2.1. Patient's Clinical Findings

SCA2 is an autosomal, dominantly inherited neurodegenerative disorder, mainly characterized by cerebellar ataxia, cerebellar atrophy at MRI, and slow eye movements [34]. LOCA is a group of patients with degenerative, genetically unrecognized pure cerebellar ataxia associated with atrophy limited to the cerebellum at MRI. Demographic, clinical, and genetic data of a larger sample including the patients recruited here have been reported by Klockgether, Muzaimi et al., and us [35–38].

### 2.2. Experiment Design

We used a highly cognitive demanding task, namely, the trail making test [39, TMT], in which subjects were asked to follow an alphanumeric sequence with their gaze. The trail making stimulus was a pop-up high contrast image consisting of a sequence of numbers and letters (1-*A*-2-*B*-3-*C*-4-*D*-5-*E*) arranged in an unpredictable manner. In our version of the trail making task (TMT), numbers were at the bottom and letters at the top of the image.

The TMT version proposed was simplified to help subjects to perform the task efficiently and avoiding any performance requirement (In the current experiment we enrolled patients affected by cerebellar disease; psychological test proposed to patients could be biased by subjective self-underestimation, fear of judgment, or fatigue; therefore, we used a simplified easy version to avoid eliciting of frustration). The distribution of symbols in a predefined geometric order allowed a more clear definition of the gaze shift during sequencing, since the distance from the center and between the symbols required a real gaze shift avoiding the target detection by periphery [40, 41].

During the task, the subject was asked to fixate a central red dot; after 500 ms the dot disappeared, and the subject could explore the TMT (Figure 2). The TMT is particularly suitable for studying selective attention, as it does not require any explicit feedback by subjects, and the test performance can be evaluated automatically and reproduced by a computational model.

#### 2.2.1. Subjects Enrollment and Training

Seven SCA2 patients, a mixed group of six patients with genetic cerebellar ataxia (LOCA), and 23 healthy subjects were enrolled in the study. All were in the age range of 25–55 years.

The patients included in the study were previously diagnosed as reported by Federighi et al. [37]. Exclusion criteria for control subjects included any history of neurological or eye problems, toxic or drug abuse, and current pharmacological treatment for neurological or eye diseases. All subjects gave their informed consent and the study was approved by the Regional Ethics Committee.

All subjects were trained by a psychologist, before the experiment, showing a paper version of the TMT. All subjects performed a first attempt of the experiment for 30 seconds; after a pause of five minutes the procedure started.

#### 2.2.2. TMT Procedure

Subjects were seated at a viewing distance of 78 cm from a 32′ color monitor (51 cm × 31 cm = 33.2 deg × 21.6 degree of visual angle). Eye position was recorded using an ASL 6000 system, which consists of a remote-mounted camera sampling pupil location at 240 Hz. Head movements were restricted using chin rest and bite. After the calibration phase, subjects performed a simple validation phase eliciting four reflexive saccades and measuring the error between the eye position and the target; the calibration procedure was repeated until the error was less than 0.5 degree. A red dot appeared at the screen center for five seconds; then a randomized version of TMT (different from the version used during the training step) appeared for 30 seconds. Subjects could stop the experiment if they thought they had concluded. All subjects performed the experiment within the given time. Different sessions of the experiment were performed for each patient on the same day as well as on different days. When a patient asked to stop the experiment because he/she was tired, the procedure was restarted after a resting period. All subjects were able to perform the task. Seven patients reported fatigue.

#### 2.2.3. Patient's Test Postassessment

To verify the visual acuity and the ability to perform the test by patients, we implemented a “guided TMT search”: a grayscale TMT version was proposed to patients (SCA2, LOCA) where letters and numbers were highlighted by a red color step-by-step following the alphanumeric sequence (1-*A*-2-*B*-3-*C*-4-*D*-5-*E*) every 2 seconds. We measured the sequencing ability and the distribution of eye fixations. Indeed, some other studies [37, 42–45] reported that cerebellar lesions injury may affect the accuracy of saccades.

### 2.3. Distribution of Eye Fixations Evaluation

To analyze visual search and model outcome, we defined some indicators. For each target (letter and number), we defined a region of interest (ROI) (100 px × 100 px). ROI was defined as ≈200% larger than the symbols of the test. We evaluated how humans directed next exploration according to the distribution of latest fixations (see Section 2.4). The fixations were identified by the dispersion algorithm developed by Salvucci and Goldberg [46].

**Figure 3:**
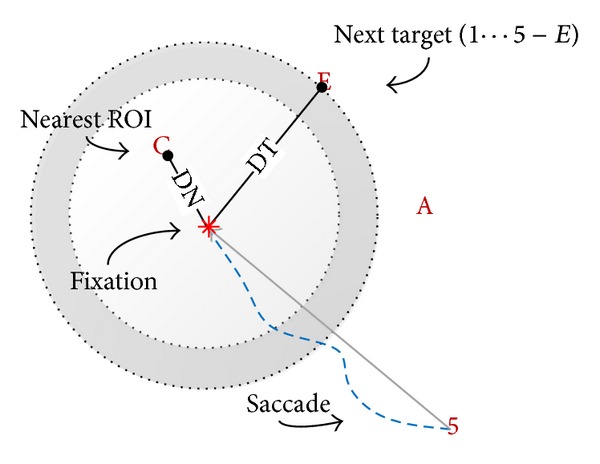
Fixations distribution indicators. For each fixation, the euclidean distance from the center of nearest ROI (DN) and the euclidean distance in pixels from the center of target (DT) were evaluated. Dashed blue line represents the eye movement (saccade).

For each fixation, we evaluated the Euclidean distance in pixels from the center of nearest ROI (DN) and the euclidean distance in pixels from the center of target (DT) (see Figure 3).

### 2.4. Selection Mechanism Evaluation

**Figure 4:**
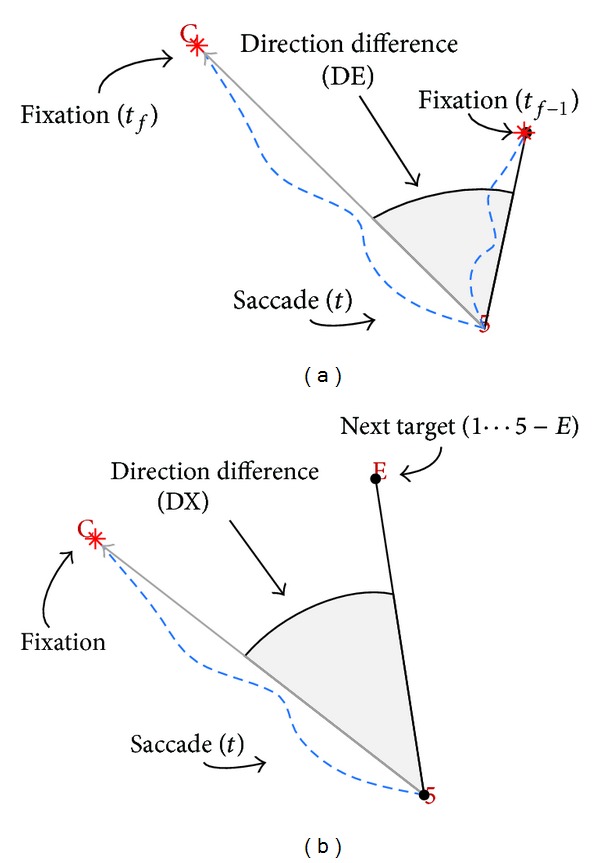
Projection of gaze: the method evaluates the difference from the direction taken by subject (saccade, dashed blue line) with respect (DE, DX) to a direction reference. (a) Direction reference was defined for DEas the vector to the previous fixations made in a given time interval (on figure only one fixation is shown) and (b) the expected target for DX.

To evaluate ongoing visual search Engel, Ponsoda et al., Findlay et al. [5, 47, 48], and, later, us Veneri et al. [41] developed a geometric method (Figure 4); the proposed procedure defined “observed direction” as the direction of the subject's gaze. We evaluated the

(1) $$\begin{array}{c} \;d=\left(\text{observed}\;\text{direction}⊖\text{direction}\;\text{reference}\right)\cdot w. \end{array}$$

*w*  is a special weight avoiding artifact due to borders; for example, the weight (*w*) was set to 1 for letter in the center and 1.6 = 8/5 for letter near the border. The ⊖ operator is the radial difference between the two directions and ranged from 0  deg⁡ to *π*  deg⁡.

The selection mechanism was evaluated calculating the direction of saccade at given time *t* (Figure 4(a)) versus the previous fixations made (DE) in a given time interval and the direction of saccade versus the expected target (DX) (Figure 4(b)).

Then, setting the “direction reference” as the direction from the current fixation to the previous fixation at the given time *t*_*f*_, the scalar direction difference DE was expressed as

(2) $$\begin{array}{c} \text{DE}=\text{mean}\left(d\right). \end{array}$$

We defined also

(3) $$\begin{array}{c} \text{DE}\left(\Delta t\right)=\text{mean}\left(d\right)\;\forall \;\text{fixations}\;\in \left(t_{f}-\Delta t,t_{f}\right). \end{array}$$

Finally, setting the “direction reference” as the direction from the current fixation to the expected direction (the direction to target) at a given time *t*_*f*_, the scalar direction difference DX was expressed as

(4) DX= mean((direction  observed⊖direction  target  expected)·w).

DX models the ability of humans to remember visited ROI and must be calculated.

### 2.5. Calculation

**Figure 5:**
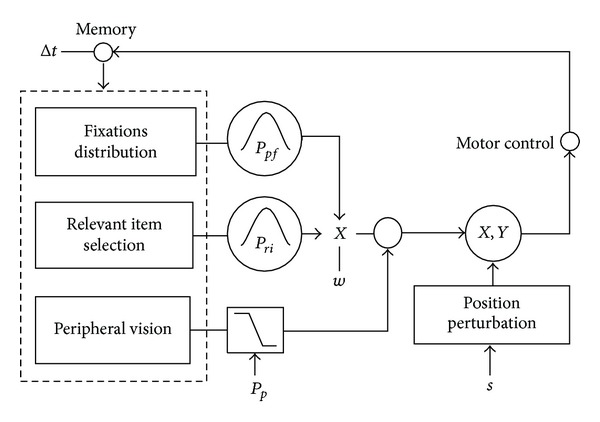
Stochastic model architecture: the block “fixations distribution” provided the probability of moving the gaze far from the latest fixations; the block “relevant item selection” provided the probability of directing the gaze to the correct target (next symbol) based on an internal representation of the target; “peripheral vision” directed the gaze to target when target is near 115 px ≈ 4 degree with probability *P*_*p*_. Model perturbation was accomplished through the *s* parameter.

Figure 5 shows the model architecture. The model was based on three subsystems: the first block (“relevant item selection” of Figure 5) provided the probability of directing the gaze to the correct target (next symbol) based on an internal representation of the target with probability *P*_*ri*_; the second block “fixations distribution” of Figure 5 provided the probability of moving the gaze far from the latest fixations with probability *P*_*pf*_ [41, 49, 50]. Third block (“peripheral vision”) directed the gaze to target when target was in a neighborhood of 115 px ≈ 4 degree with probability *P*_*p*_ and modeled covert attention [19]. Latest block perturbed (*s*) fixations location in order to simulate model variability.

After normalization, the weighted union probability between two mutually exclusive events was calculated (5):

(5) $$\begin{array}{c} P\;\text{next}\;\text{target}\;=\left(1-w\right)\cdot P_{ri}\left(\text{DX},\sigma_{ri}^{2}\right)+w\cdot P_{pf}\left(\text{DE},\sigma_{pf}^{2}\right), \end{array}$$

where *w* ∈ (0,1).

The model calculated the probability by (5): then it selected the direction to move in accordance with the probability. The procedure was repeated for each symbol and exited when the model performed the sequence correctly. *w* was the level of competition between the blocks “relevant item selection” and “fixations distribution.”

Probabilities are *P*_*ri*_ = *𝒩*(DX, *σ*_*ri*_^2^), *P*_*pf*_ = *𝒩*(DE, *σ*_*pf*_^2^), and *P*_*p*_ = *𝒩*(*μ*_*p*_, 1), where *𝒩*(*μ*, *σ*) is the normal distribution of mean *μ* and variance *σ*. DE and DX were evaluated through (2) and (4).

Output of model was an array of fixations

(6) $$\begin{array}{c} \left(x_{f},y_{f}\right)=\Phi \left(s,\mu_{p},w;\sigma_{pf}^{2},\sigma_{ri}^{2}\right), \end{array}$$

where *σ*_*pf*_^2^ was set to 2, according to variance of DE of subjects, *σ*_*ri*_^2^ was set to 3.4, according to variance of DX of subjects, *s*, and *μ*_*p*_ and *w* were free variables.

### 2.6. Energy Function

Optimization theory requires the definition of one (or more) energy/cost function to be minimized. Therefore, according to [45, 51, 52], we defined two function costs based on saccades' properties; for each saccade, we evaluated the euclidean distance in pixels from the saccade start point to the end of saccade (*A*_sacc_), skipping short saccade inside the same ROI. A global function saccade energy was measured evaluating the path length through the following formulas:

(7) Jsacc =∑∀t∈saccades(x(t)−x(t−δt))2+(y(t)−y(t−δt))2,

where *δt* = 4.167 ms is the sampling time. Equation (7) is the sum of all saccades' length.

Fixations represent the cognitive act to process the scene; cardinality and duration are measures of task performance [19, 53, 54]. Therefore, the task execution energy was defined counting the number of fixations inside the ROI to complete the task:

(8) $$\begin{array}{c} J_{\text{fix}}=\overset{}{\underset{\forall \text{fixations}\;\text{in}\;\text{ROI}}{\sum }}1. \end{array}$$

Equation (8) is the number of steps made to complete the task.

### 2.7. Stochastic Model Application

The basic idea was to apply the stochastic model defined in Section 2.5 to study some target function (energy *J*_sacc_, *J*_fix_, and *A*_sacc_) as an optimal control problem. Optimization problems can mostly be seen as one of two kinds: we need to find the extrema of a target function cost over a given domain; performance is highly dependent on the analytical properties of the target function. Therefore, if the target function is too complex to allow an analytical study or if the domain is too irregular, the method of choice is rather the stochastic approach [55, 56]. Since visual search is a complex system under the influence of many mechanisms, it is not easy to predict the selection mechanism through an implicit and deterministic model; therefore, we developed a stochastic model based on the MC simulation (To understand Monte Carlo simulation the reader should consider the following case: a player wants to measure the surface of his carpet in his room 3 m × 3 m; the player may randomly launch a button 100 times and count the number of times (*k*) the button falls onto the carpet. It is easy to verify that the carpet surface is *k* · (3 m × 3 m)/100. In our work, the sought-after measure is the energy to execute the task (the carpet surface) and the sought after system is the set of parameters (the carpet geometry).) [57]. Therefore, the model attempted all possible explorative strategies, varying parameters *s*, *μ*_*p*_, and *w*. For each *n* = 1000 simulations, we varied parameters and we evaluated the function cost *J*_sacc_, *A*_sacc_, and *J*_fix_ (Monte Carlo optimization).

**Figure 6:**
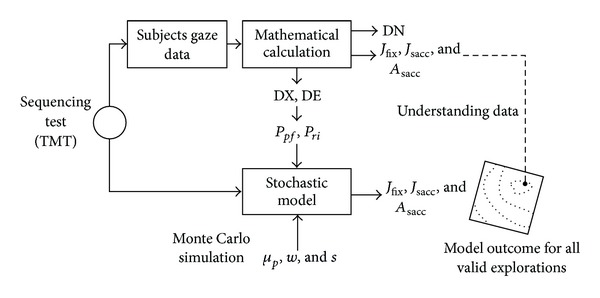
Analysis procedure. Subjects' gaze data were evaluated through a mathematical procedure able to extract some basic indicators (DN, DX, and DE) and energy functions (*J*_sacc_, *A*_sacc,_ and *J*_fix_). DX and DE were used to calculate variance (*σ*_*pf*_^2^, *σ*_*ri*_^2^) to tune the model. Then, using varying parameters *s*, *μ*_*p*_, and *w*, model reproduced all valid explorations. Subjects' and model's outcomes were compared to understand the selection mechanism.

From an intuitive point, the model computed the solutions domain perturbing fixations distribution; the outcome could be compared with subjects' visual search (CTRL, LOCA, and SCA2) data; see Figure 6.

## 3. Results

### 3.1. Subjects

**Figure 7:**
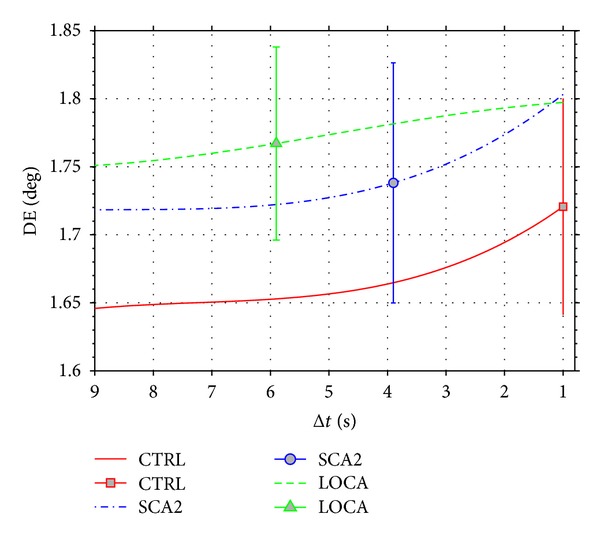
Direction difference trend of subjects. The method calculated the direction difference DE from previous fixations made on the time intervals [0 ⋯ 9], [0 ⋯ 8], ⋯, [0 ⋯ 1] seconds (on the *x*-axis only the upper interval's value is reported). The figure shows the standard deviation of CTRL, LOCA, and SCA2 for Δ*t* = 1,4, 6, respectively. The positive trend of the lines shows that saccade's direction tended to move away from latest fixations.

The positive trend of DE(Δ*t*) − DE (Figure 7) for all groups suggested that saccade's direction tended to move away from latest fixations. In particular, the trend of gaze direction with respect to the distribution of fixations made increased in the last second (Δ*t* = 1 s) suggesting that the basic model operation (Section 2.5) was compatible with subjects' exploration.

To assess the differences of exploration strategy among groups, we evaluated distance to nearest ROI (DN) and number of visited regions of interest (*J*_fix_). ANOVA did not report significant difference on *J*_fix_ (*P* = 0.126, *F*(2,33) = 2.209) and it was confirmed by posthoc analysis (*p*_CTRL-SCA2_ = 0.6596, *p*_CTRL-LOCA_ = 0.0653, and *p*_SCA2-LOCA_ = 0.0641). On the contrary, ANOVA reported a significant difference on DN (*P* = 0.001, *F*(2,33) = 9.52) and post-hoc Holm-Sidak confirmed the significant difference of DN CTRL-SCA2 (*p*_CTRL-SCA2_ < 0.001, *α*(33) = 0.0170) and between CTRL-LOCA (*p*_CTRL-LOCA_ = 0.0105, *α*(33) = 0.0253) and no significant difference between patients (*p*_SCA2-LOCA_ = 0.4217, *α*(33) = 0.0500).

Our preliminary conclusion was that performance could be considered equivalent among groups but with different strategies (Table 1). Indeed, patients (LOCA and SCA2) preferred sparser fixations instead of targeted saccades. This strategy was found by several authors [58–60] and has been referred fixation as “center-of-gravity” fixations. Center-of-gravity occurs when targets are surrounded by nontargets, and the saccades, instead of landing at the designated target, land in the midst of the whole configuration. This effect was, firstly, attributed to an error of the filter selection [61] and then was considered a necessary mechanism to execute more efficient saccades [60, 62]. We concluded that patients could direct the gaze into the ROI, but preferred sparse fixations. To understand this effect, we compared human results with model results.

### 3.2. Model Simulation

**Figure 8:**
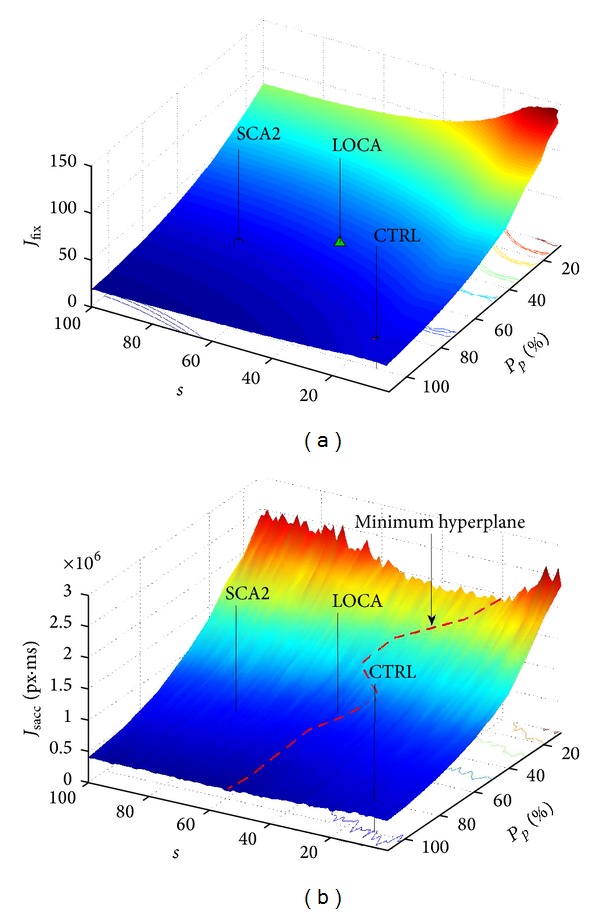
Results of the model varying parameters. (a) The number of steps to complete the task by model and by subjects. The surface reports the domain of all available explorations of model that humans could perform. Stressing covert attention humans could perform the best exploration (“center-of-gravity” fixations), but similar performance could be gained directing the gaze to an intermediate region to acquire overall scene information; this strategy was preferred by SCA2 and LOCA. (b) Saccade energy spent by the model for all available explorations that humans could perform. An overall minimum hyperplane was found at *s* ≈ 32 ± 8.2.

Using varying parameters *s*, *μ*_*p*_, and *w*, the model (Figure 5) calculated the *J*_fix_, *J*_sacc_, and *A*_sacc_: the three surfaces (slightly smoothed for readability) shown in Figures 8 and 10, report the domain of all available explorations choosing the minimum value along the dimension of the parameter *w*.

To assess the validity of the model we evaluated the normalized root mean square error (NRMSD) of *J*_fix_. NRMSD varied from 9% to 26%: NRMSD_CTRL_ = 0.091, NRMSD_LOCA_ = 0.17, and NRMSD_SCA2_ = 0.26. We accepted this result as an acceptable error; indeed, only 11% of subjects reported a NRMSD > 0.20 and, in any case, NRMSD < 0.40.

The model reported a local minimum *J*_fix_ = 19, when *s* = 100, *μ*_*p*_ = 100, and *w* = 0.6; subjects, however, performed a less efficient exploration (Table 1). To study this result, in depth, we calculated the *J*_sacc_ parameter, which provided an estimate of saccadic energy; we have to note that it is not possible to compare *J*_sacc_ of subjects and model due to noise on saccade's trajectory; indeed, in two recent papers [37, 38], we found that motor control noise of SCA2 reported a significant difference with CTRL and LOCA.  Figure 8(b) shows the overall solutions domain varying parameters *s*, *μ*_*p*_, and *w*. Overall minimum hyperplane was found at *s* ≈ 32 ± 8.2, which corresponded to DN ≈ 60.

**Figure 9:**
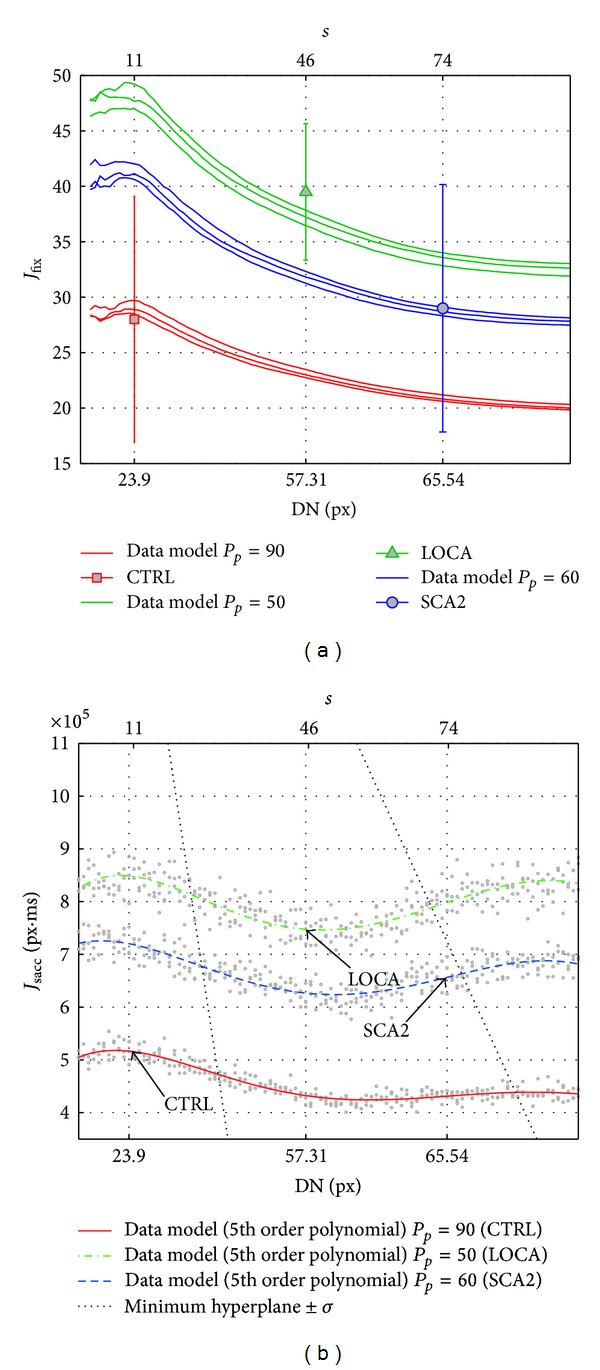
The graph reports the numbers of steps performed by model varying *s*, *μ*_*p*_, and *w*; the *s* parameter (upper axis) controlled the dispersion of fixations with direct (nonlinear) influence on DN (bottom axis),   *μ*_*p*_ set *P*_*p*_ of subjects, and *w* provided the needful variability to adapt within group variability among subjects. (a) The number of steps (*J*_fix_) to complete the task of subjects and model. (b) Saccade energy (*J*_sacc_) to complete the task of subjects and model.

Comparison of model simulations with subjects' performance was done by setting model's *μ*_*p*_ equal to *P*_*p*_ of subjects (0.90, 0.45, and 0.60); Figure 9 shows the model compared to subjects' exploration: the three groups of lines of Figure 9(a) showed the model numbers of steps to complete the task (*J*_fix_) for *μ*_*p*_ = 0.90, *μ*_*p*_ = 0.45, and *μ*_*p*_ = 0.60 and varying *s* and *w*. The *s* parameter controlled the dispersion of fixations with direct (nonlinear) influence on DN, and *w* provided the needful variability to adapt within group variability among subjects. The three lines of Figure 9(b) show the saccade energy (*J*_fix_) of the model and the corresponding value of subjects.

To evaluate the influence of saccade control on visual search, we analyzed saccade amplitude (*A*_sacc_): ANOVA reported a significant difference among groups (*P* = 0.0037, *F*(2,33) = 6.64) and post-hoc holm-sidak confirmed the significant difference of *A*_sacc_ between CTRL-SCA2 (*p*_CTRL-SCA2_ < 0.001, *α*(33) = 0.0170) and CTRL-LOCA (*p*_CTRL-LOCA_ < 0.01, *α*(33) = 0.0253) and no significant difference between patients (*p*_SCA2-LOCA_ = 0.5769, *α*(33) = 0.050). Indeed, we found that saccade amplitude of patients (SCA2 and LOCA) was less than 23.5% and 28.1% of healthy subjects' saccades (Table 1).

**Figure 10:**
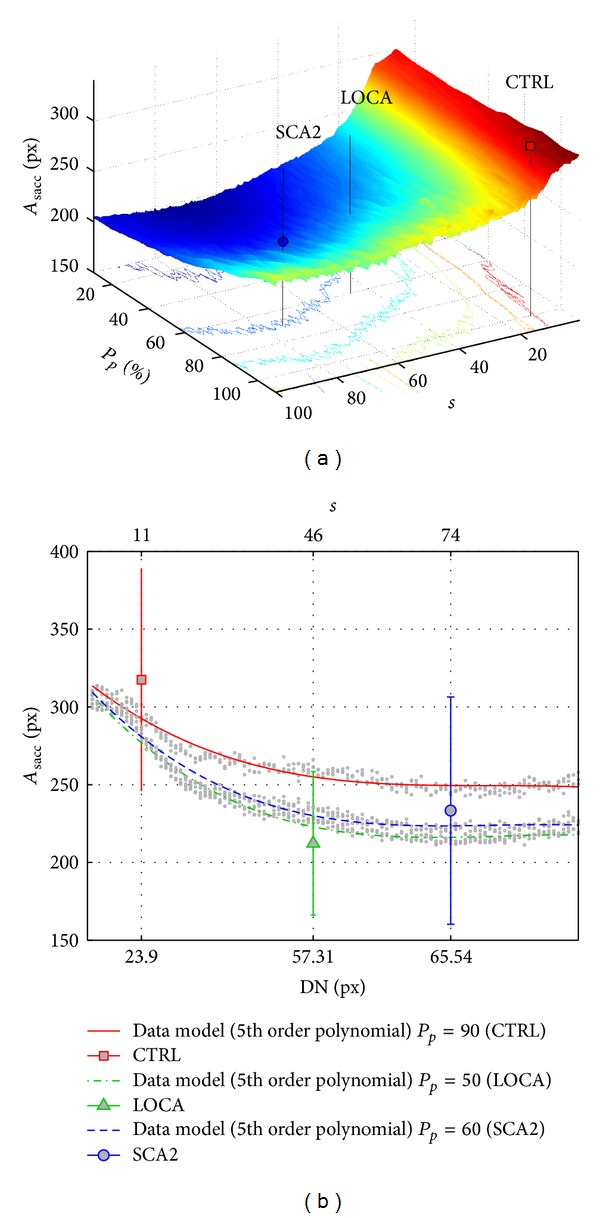
Mean saccade amplitude reported by model. (a) Overall space of saccade amplitude (*A*_sacc_) reported by model varying parameters and (b) compared with subjects' performance. Model reported that patients (SCA2 and LOCA) preferred a visual search exploration in order to reduce *A*_sacc_. ANOVA confirmed a significant difference of *A*_sacc_ between CTRL and patients.

Comparing *A*_sacc_ of subjects and model, model reported similar value (Figure 10(a)) to subjects with an error rate of ≈7%; analyzing the *A*_sacc_ trend varying fixations' dispersion (DN), it seems plausible that SCA2 and LOCA preferred an exploration which minimized saccade amplitude. We tried to minimize the following empirical function cost bringing together the goal parameter *A*_sacc_, *J*_sacc_, and *J*_fix_

(9) h(θ0,θ1,θ2) =θ0·Asaccmax⁡(Asacc)+θ1·Jsaccmax⁡(Jsacc)+θ2·Jfixmax⁡(Jfix).

We found that SCA2 and LOCA preferred to minimize saccade amplitude (*θ*_0_ = 1) and saccade energy (*θ*_1_ ≈ 1.3) rather than steps to complete the task (*θ*_2_ ≈ 0.1); the opposite was true for CTRL (*θ*_0,1,2_ = 1,0, 5.29).

### 3.3. Patients Test Postassessment Results

Since subjects with cerebellar injury have reported low precision on visual search tasks, we asked patients (SCA2, LOCA) to perform a “guided TMT search” where letters and numbers were highlighted in red step-by-step. Patients were able to complete the sequence with few fixations outside the ROI (DN = 3.01). We concluded that the low precision performance reported by several authors [37, 42, 45] was negligible compared to the size of the ROI. Similar findings were reported by van Beers.

## 4. Discussion

Assuming an error rate *≈*14% (NRMSD for *J*_fix_) of the model, both patients and healthy subjects performed a suboptimal exploration with different strategies: trend reported in Figure 9(b) suggested that patients (SCA2, LOCA) preferred sparser fixations at an intermediate position between the targets (“center-of-gravity” fixations) and optimally tuned to keep saccade amplitude (*A*_sacc_) as low as possible and energy saccade (*J*_sacc_) in a neighborhood of the minimum (suboptimal exploration); on the contrary, healthy subjects (CTRL) preferred saccades directed near targets (target selection). Performance (*J*_fix_) of SCA2 patients was similar to healthy subjects (CTRL); LOCA patients completed the task with more energy and steps but not with a significant difference from CTRL.

These different strategies on visual search exploration between healthy subjects (CTRL) and cerebellar patients (LOCA and SCA2) suggested a direct or indirect influence of the cerebellum on the visual selection processing.

### 4.1. Theoretical Considerations about the Cerebellum's Role

Traditional views of the cerebellum hold that this structure is engaged in the control of action with a specific role in the motor skills [63]. The properties of cerebellar efferent and afferent projections (see Figure 1(b) for a short reference) suggest that the cerebellum is generally involved in (“not necessarily” [64]) integrating motor control and sensory information to coordinate movements. The model of neuronal circuitry of the cerebellum proposed by Ito [27, 32, 65] makes it possible to consider more concrete ideas about cerebellar processing: cerebellum is thought to encode internal models that reproduce the dynamic properties of body parts. These models control the movement allowing the brain to precisely predict the consequence of a movement without the need for sensory feedback [66–68] or to perform a quick movement in the absence of visual feedback [27]. Indeed, cerebellum is able to reproduce a stereotyped function of the desired displacement of eyes and provides a reference signal during movement [69]. To explain this mechanism two models have been proposed. Kawato et al. [26] proposed a forward model (cerebellum forward model) that simulates the dynamics (or kinematics) of the controlled object including the lower motor centers and motor apparatus (Figure 1(b)); the motor cortex should be able to perform a precise movement using an internal feedback from the forward model instead of the external feedback from the real control object [65, 67]. As such, motor learning can be considered to be a process by which the forward model is formed and reformed in the cerebellum through based learning. When the cerebellar cortex operates in parallel with the motor cortex, as mentioned above, it forms another type of internal model that bears a transfer function, which is reciprocally equal to the dynamics (or kinematics) of the control object [25, 26]. The inverse model can then play the role of a feedforward controller that replaces the motor cortex serving as a feedback controller.

As further proof of this theory, it is well known that cerebellar lesions [43, 44] induce permanent deficits, affecting dramatically the consistency [42, 45] and the accuracy of saccades [37]. While a wide range of evidence has emerged accounting for a dominant role of cerebrocerebellar interactions in motor control and its movement-related functions are the most solidly established that, recent studies have clearly suggested an influence of the cerebellum in cognitive and behavioral functions [65, 67, 70, 71], including fear and pleasure responses [72–74]. Allen et al. [75], through a magnetic resonance experiment, found a direct activation, during visual attention, of some cerebellar areas independent from those deputed to motor performance. Paulin [64] developed a computational physical model estimating the movement status, useful for trajectory tracking and trajectory prediction; Paulin concluded that the cerebellum is not a motor control device *per se* but a device for optimizing sensory information about movements. Kellermann et al. [76] identified a cerebelloparietal loop consisting of posterior parietal cortex (visual area V5, cerebellum) that facilitates predictions of dynamic perceptual events. Other studies provided findings about a direct implication of the cerebellum on language, verbal working memory [77–81], and timing [82]. Gottwald et al. reported significant defects of patients with focal cerebellar lesions in the divided attention and working memory but not in selective attention task [83]. In addition, Exner et al. [84], Golla et al. [85], and later Hokkanen et al. [86] reported normal attentional shifting in patients affected by cerebellar lesions.

### 4.2. The Dominant Role of the Cerebellum

A number of functional hypotheses (sometimes contradictories [87]) have been advanced to account for how the cerebellum may contribute to cognition and a large amount of neuroanatomical studies showed cerebellar connectivity with almost all the associative areas of the cerebral cortex involved in higher cognitive functioning [73]; nevertheless, the hypothesis of the dominant role of the cerebellum on motor control remains the most probable to explain our findings and was confirmed by the proposed model. Cerebellar patients have a well-known disturbance in controlling saccade endpoint error. The two groups reported here, however, substantially show a different saccadic behavior, due to the involvement of diverse anatomic structures. The saccadic behavior mainly characterizing SCA2 is the low speed with quite preserved accuracy indicating a failure of the brainstem saccade generator more than cerebellum. LOCA shows a well-preserved speed but a complete loss of saccadic error control. This pathophysiological substrate, however, does not distinguish their visual exploration during sequencing; indeed, both groups of patients similarly performed a multi step visual search avoiding the direct foveation of the target. This multistep spatial sampling strategy probably enables these patients to increase the discriminative potentialities of the covert attention avoiding unnecessary large saccades moving the eyes over wrong targets. If this strategy is true, saccades not only are associated with an overt shift of attention but also increase the potentialities of covert attention during active search. Moreover, when the cerebellum has reduced capacities, the control of long saccade is difficult, due to propagation of error; [51] and [45] found that patients affected by SCA2 reduced saccade velocity in reflexive tasks (involuntary movement); Rufa and Federighi [88] found that SCA2 patients, sometimes, interrupted saccades. Then, it is plausible that in voluntary movements (free visual search test) patients, affected by cerebellar disease, adapted visual search exploration to minimize the saccade control effort; they preferred sparse fixations and short saccades, but maintained overall saccade energy around a minimum point. The implemented model supported this hypothesis.

## 5. Conclusions

In our work, we implemented a stochastic model able to replicate humans strategies during an ongoing sequencing test; the model results were compared to the performance of a group of healthy subjects and two groups of patients having well-documented motor control disease.

From the methodological point of view, we introduced the optimization method (OCT) to study the properties of selective attention. Todorov and later van Beers proposed the OCT as a valuable instrument to link eye/hand motor control and the central nervous system to minimize the consequence of motor control noise. Najemnik and Geisler implemented a Bayesian framework to validate that human visual search is a sophisticated mechanism that maximizes the information collected across fixations. We “connected” these two theories to integrate the motor control and information-processing systems on a single reproducible model. Our conclusions are similar to the conclusion of Najemnik and Geisler: humans tend to tune visual search in order to maximize information collected during the search, but, in clinical context, patients try to minimize the effort to control eye movements.

Therefore, our opinion is that humans tend to apply an optimal selection to minimize a function cost (effort, energy), and we provided evidence of this theory studying the motor control influence through a model based on MC optimization method and comparing results with cerebellar patients. Proposed model, however, is affected by some restrictions: model is specific for the TMT test and it is not easy to generalize to other psychological tests; function costs have to be defined according to the disease studied. In our future work, we aim to adapt the basic principle of OCT and MC on the real image processing model.

## Figures and Tables

**Table 1:** Mean value and standard deviation (in brackets) of tests.

Indicators	Acronym	CTRL	LOCA	SCA2
Group dimension	*N*	23	6	7
Age		27–50	40–55	42–55
Distance to nearest ROI (px)	DN	23.9 (14.35)	57.31 (±13.68)	65.54 (±18.49)
Number of fixations in ROI (visited ROI)	*J*_fix_	28.90 (±11.18)	37.5 (±6.15)	29.2 (±11.16)
Number of fixations		39	68.1	49.3
Saccade amplitude (px)	*A*_sacc_	317.36 (±69.64)	212.22 (±46.10)	233.32 (±73.05)
Within subject saccade amplitude variance		49634 (±21329)	24040 (±6200)	33138 (±16031)
Percentage of saccade directed to target when DT ≤ 4 degree (%)	*P*_*p*_	0.90	0.45	0.60

## References

[1] Posner MI, Driver J (1992). The neurobiology of selective attention. *Current Opinion in Neurobiology*.

[2] Findlay JM, Gilchrist ID (2003). *Active Vision: The Psychology of Looking and Seeing*.

[3] Siegel M, Körding KP, König P (2000). Integrating top-down and bottom-up sensory processing by somato-dendritic interactions. *Journal of Computational Neuroscience*.

[4] Breitmeyer BG, Kropfl W, Julesz B (1982). The existence and role of retinotopic and spatiotopic forms of visual persistence. *Acta Psychologica*.

[5] Findlay JM, Brown V, Gilchrist ID (2001). Saccade target selection in visual search: the effect of information from the previous fixation. *Vision Research*.

[6] Haji-Abolhassani A, Clark JJ (2013). A computational model for task inference in visual search. *Journal of Vision*.

[7] Fecteau JH, Munoz DP (2006). Salience, relevance, and firing: a priority map for target selection. *Trends in Cognitive Sciences*.

[8] Carrasco M, McElree B (2001). Covert attention accelerates the rate of visual information processing. *Proceedings of the National Academy of Sciences of the United States of America*.

[9] Roska B, Molnar A, Werblin FS (2006). Parallel processing in retinal ganglion cells: how integration of space-time patterns of excitation and inhibition form the spiking output. *Journal of Neurophysiology*.

[10] Wurtz RH, McAlonan K, Cavanaugh J, Berman RA (2011). Thalamic pathways for active vision. *Trends in Cognitive Sciences*.

[11] Leopold DA (2012). Primary visual cortex, awareness and blindsight. *Annual Review of Neuroscience*.

[12] Li Z (2002). A saliency map in primary visual cortex. *Trends in Cognitive Sciences*.

[13] Krauzlis RJ, Lovejoy LP, Zenon A (2013). Superior colliculus and visual spatial attention. *Annual Review of Neuroscience*.

[14] Schneider GE (1969). Two visual systems. *Science*.

[15] Corbetta M, Shulman GL (2002). Control of goal-directed and stimulus-driven attention in the brain. *Nature Reviews Neuroscience*.

[16] Connor CE (2003). Active vision and visual activation in area v4. *Neuron*.

[17] McIntosh RD, Schenk T (2009). Two visual streams for perception and action: current trends. *Neuropsychologia*.

[18] Reynolds JH, Heeger DJ (2009). The normalization model of attention. *Neuron*.

[19] Carrasco M (2011). Visual attention: the past 25 years. *Vision Research*.

[20] Treisman AM, Gelade G (1980). A feature-integration theory of attention. *Cognitive Psychology*.

[21] Wolfe JM (1994). Guided search 2.0-a revised model of visual-search. *Psychonomic Bulletin & Review*.

[22] Rizzolatti G, Riggio L, Dascola I, Umilta C (1987). Reorienting attention across the horizontal and vertical meridians: evidence in favor of a premotor theory of attention. *Neuropsychologia A*.

[23] Bundesen C, Habekost T, Kyllingsbæk S (2005). A neural theory of visual attention: bridging cognition and neurophysiology. *Psychological Review*.

[24] Itti L, Koch C (2000). A saliency-based search mechanism for overt and covert shifts of visual attention. *Vision Research*.

[25] Ito M (1984). The modifiable neuronal network of the cerebellum. *Japanese Journal of Physiology*.

[26] Kawato M, Furukawa K, Suzuki R (1987). A hierarchical neural-network model for control and learning of voluntary movement. *Biological Cybernetics*.

[27] Ito M (2005). Bases and implications of learning in the cerebellum- adaptive control and internal model mechanism. *Progress in Brain Research*.

[28] Todorov E (2005). Stochastic optimal control and estimation methods adapted to the noise characteristics of the sensorimotor system. *Neural Computation*.

[29] van Beers RJ (2008). Saccadic eye movements minimize the consequences of motor noise. *PLoS ONE*.

[30] Osborne LC (2011). Computation and physiology of sensory-motor processing in eye movements. *Current Opinion in Neurobiology*.

[31] Najemnik J, Geisler WS (2008). Eye movement statistics in humans are consistent with an optimal search strategy. *Journal of Vision*.

[32] Kelly RM, Strick PL (2003). Cerebellar loops with motor cortex and prefrontal cortex of a nonhuman primate. *Journal of Neuroscience*.

[33] Zee DS, Leigh RJ (2006). *The Neurology of Eye Movements (Book/DVD)*.

[34] Restivo DA, Giuffrida S, Rapisarda G (2000). Central motor conduction to lower limb after transcranial magnetic stimulation in spinocerebellar ataxia type 2 (SCA2). *Clinical Neurophysiology*.

[35] Klockgether T (2000). *Handbook of Ataxia Disorders*.

[36] Muzaimi MB, Thomas J, Palmer-Smith S (2004). Population based study of late onset cerebellar ataxia in south east Wales. *Journal of Neurology, Neurosurgery and Psychiatry*.

[37] Federighi P, Cevenini G, Dotti MT (2011). Differences in saccade dynamics between spinocerebellar ataxia 2 and late-onset cerebellar ataxias. *Brain*.

[38] Veneri G, Federighi P, Rosini F, Federico A, Rufa A (2011). Spike removal through multiscale wavelet and entropy analysis of ocular motor noise: a case study in patients with cerebellar disease. *Journal of Neuroscience Methods*.

[39] Bowie CR, Harvey PD (2006). Administration and interpretation of the trail making test. *Nature Protocols*.

[40] Veneri G, Pretegiani E, Rosini F, Federighi P, Federico A, Rufa A (2012). Evaluating the human ongoing visual search performance by eye tracking application and sequencing tests. *Computer Methods and Programs in Biomedicine*.

[41] Veneri G, Rosini F, Federighi P, Federico A, Rufa A (2012). Evaluating gaze control on a multi-target sequencing task: the distribution of fixations is evidence of exploration optimisation. *Computers in Biology and Medicine*.

[42] Zee DS, Optican LM, Cook JD (1976). Slow saccades in spinocerebellar degeneration. *Archives of Neurology*.

[43] Optican LM, Robinson DA (1980). Cerebellar-dependent adaptive control of primate saccadic system. *Journal of Neurophysiology*.

[44] Lefèvre P, Quaia C, Optican LM (1998). Distributed model of control of saccades by superior colliculus and cerebellum. *Neural Networks*.

[45] Ramat S, Leigh RJ, Zee DS, Optican LM (2007). What clinical disorders tell us about the neural control of saccadic eye movements. *Brain*.

[46] Salvucci DD, Goldberg JH Identifying fixations and saccades in eye-tracking protocols.

[47] Engel FL (1977). Visual conspicuity, visual search and fixation tendencies of the eye. *Vision Research*.

[48] Ponsoda V, Scott D, Findlay JM (1995). A probability vector and transition matrix analysis of eye movements during visual search. *Acta Psychologica*.

[49] Klein RM (2000). Inhibition of return. *Trends in Cognitive Sciences*.

[50] Foti F, Mandolesi L, Cutuli D (2010). Cerebellar damage loosens the strategic use of the spatial structure of the search space. *Cerebellum*.

[51] Zee DS, Leigh RJ (2006). *The Neurology of Eye Movements (Book/DVD)*.

[52] Matsumoto H, Terao Y, Furubayashi T (2012). Basal ganglia dysfunction reduces saccade amplitude during visual scanning in Parkinson’s disease. *Basal Ganglia*.

[53] Yarbus AL (1967). *Eye Movements and Vision*.

[54] Zingale CM, Kowler E (1987). Planning sequences of saccades. *Vision Research*.

[55] Robert CP, Casella G (2009). *Introducing Monte Carlo Methods*.

[56] Kim B, Basso MA (2010). A probabilistic strategy for understanding action selection. *Journal of Neuroscience*.

[57] Metropolis N, Ulam S (1949). The Monte Carlo method. *Journal of the American Statistical Association*.

[58] Findlay JM (1982). Global visual processing for saccadic eye movements. *Vision Research*.

[59] Melcher D, Kowler E (1999). Shapes, surfaces and saccades. *Vision Research*.

[60] Cohen EH, Schnitzer BS, Gersch TM, Singh M, Kowler E (2007). The relationship between spatial pooling and attention in saccadic and perceptual tasks. *Vision Research*.

[61] Kowler E (2011). Eye movements: the past 25 years. *Vision Research*.

[62] Findlay JM, Blythe HI (2009). Saccade target selection: do distractors affect saccade accuracy?. *Vision Research*.

[63] Ramnani N (2006). The primate cortico-cerebellar system: anatomy and function. *Nature Reviews Neuroscience*.

[64] Paulin MG (2005). Evolution of the cerebellum as a neuronal machine for Bayesian state estimation. *Journal of Neural Engineering*.

[65] Ito M (2006). Cerebellar circuitry as a neuronal machine. *Progress in Neurobiology*.

[66] Barlow JS (2002). *The Cerebellum and Adaptive Control*.

[67] Ito M (2008). Control of mental activities by internal models in the cerebellum. *Nature Reviews Neuroscience*.

[68] King S, Chen AL, Joshi A, Serra A, Leigh RJ (2011). Effects of cerebellar disease on sequences of rapid eye movements. *Vision Research*.

[69] Molinari M, Filippini V, Leggio MG (2002). Neuronal plasticity of interrelated cerebellar and cortical networks. *Neuroscience*.

[70] Leiner HC, Leiner AL, Dow RS (1986). Does the cerebellum contribute to mental skills?. *Behavioral Neuroscience*.

[71] Fiez JA (1996). Cerebellar contributions to cognition. *Neuron*.

[72] Tavano A, Grasso R, Gagliardi C (2007). Disorders of cognitive and affective development in cerebellar malformations. *Brain*.

[73] Baillieux H, Smet HJD, Paquier PF, De Deyn PP, Mariën P (2008). Cerebellar neurocognition: insights into the bottom of the brain. *Clinical Neurology and Neurosurgery*.

[74] Stoodley CJ, Schmahmann JD (2010). Evidence for topographic organization in the cerebellum of motor control versus cognitive and affective processing. *Cortex*.

[75] Allen G, Buxton RB, Wong EC, Courchesne E (1997). Attentional activation of the cerebellum independent of motor involvement. *Science*.

[76] Kellermann T, Regenbogen C, De Vos M, Monang C, Finkelmeyer A, Habel U (2012). Effective connectivity of the human cerebellum during visual attention. *The Journal of Neuroscience*.

[77] Leiner HC, Leiner AL, Dow RS (1991). The human cerebro-cerebellar system: its computing, cognitive, and language skills. *Behavioural Brain Research*.

[78] Leiner HC, Leiner AL, Dow RS (1993). Cognitive and language functions of the human cerebellum. *Trends in Neurosciences*.

[79] Desmond JE, Gabrieli JDE, Wagner AD, Ginier BL, Glover GH (1997). Lobular patterns of cerebellar activation in verbal working-memory and finger-tapping tasks as revealed by functional MRI. *Journal of Neuroscience*.

[80] Ravizza SM, McCormick CA, Schlerf JE, Justus T, Ivry RB, Fiez JA (2006). Cerebellar damage produces selective deficits in verbal working memory. *Brain*.

[81] Passamonti L, Novellino F, Cerasa A (2011). Altered cortical-cerebellar circuits during verbal working memory in essential tremor. *Brain*.

[82] Hong S, Optican LM (2008). Interaction between Purkinje cells and inhibitory interneurons may create adjustable output waveforms to generate timed cerebellar output. *PLoS ONE*.

[83] Gottwald B, Mihajlovic Z, Wilde B, Mehdorn HM (2003). Does the cerebellum contribute to specific aspects of attention?. *Neuropsychologia*.

[84] Exner C, Weniger G, Irle E (2004). Cerebellar lesions in the PICA but not SCA territory impair cognition. *Neurology*.

[85] Golla H, Thier P, Haarmeier T (2005). Disturbed overt but normal covert shifts of attention in adult cerebellar patients. *Brain*.

[86] Hokkanen LSK, Kauranen V, Roine RO, Salonen O, Kotila M (2006). Subtle cognitive deficits after cerebellar infarcts. *European Journal of Neurology*.

[87] Bischoff-Grethe A, Ivry RB, Grafton ST (2002). Cerebellar involvement in response reassignment rather than attention. *Journal of Neuroscience*.

[88] Rufa A, Federighi P (2011). Fast versus slow: different saccadic behavior in cerebellar ataxias. *Annals of the New York Academy of Sciences*.

